# Phase Formation and High-Temperature Stability of Very Thin Co-Sputtered Ti-Al and Multilayered Ti/Al Films on Thermally Oxidized Si Substrates

**DOI:** 10.3390/ma13092039

**Published:** 2020-04-27

**Authors:** Marietta Seifert, Eric Lattner, Siegfried B. Menzel, Steffen Oswald, Thomas Gemming

**Affiliations:** Leibniz IFW Dresden, Helmholtzstraße 20, 01069 Dresden, Germany; e.lattner@ifw-dresden.de (E.L.); s.menzel@ifw-dresden.de (S.B.M.); s.oswald@ifw-dresden.de (S.O.); t.gemming@ifw-dresden.de (T.G.)

**Keywords:** TiAl, thin films, surface acoustic waves, high-temperature stability, phase formation

## Abstract

Ti-Al thin films with a thickness of 200 nm were prepared either by co-sputtering from elemental Ti and Al targets or as Ti/Al multilayers with 10 and 20 nm individual layer thickness on thermally oxidized Si substrates. Some of the films were covered with a 20-nm-thick SiO${}_{2}$ layer, which was used as an oxidation protection against the ambient atmosphere. The films were annealed at up to 800 °C in high vacuum for 10 h, and the phase formation as well as the film architecture was analyzed by X-ray diffraction, cross section, and transmission electron microscopy, as well as Auger electron and X-ray photoelectron spectroscopy. The results reveal that the co-sputtered films remained amorphous after annealing at 600 °C independent on the presence of the SiO${}_{2}$ cover layer. In contrast to this, the γ-TiAl phase was formed in the multilayer films at this temperature. After annealing at 800 °C, all films were degraded completely despite the presence of the cover layer. In addition, a strong chemical reaction between the Ti and SiO${}_{2}$ of the cover layer and the substrate took place, resulting in the formation of Ti silicide. In the multilayer samples, this reaction already started at 600 °C.

## 1. Introduction

Ti-Al-based materials are widely used in industry, especially for aerospace applications in aircraft engines [1] or gas turbines [2] due to their high thermal stability in combination with a low density. Therefore, much research has been performed on Ti-Al based bulk material and on Ti-Al coatings. There is also literature on Ti-Al thin films; however, in general, this refers to films with a thickness of a few or tens of μm [3,4,5]. Besides the application as bulk material or μm-thick coating, Ti-Al films with a thickness of a few 100 nm are interesting as a metallization in surface acoustic wave (SAW) devices operating at elevated temperatures (above 400 °C).

Current research on high temperature stable metallizations for SAW devices mainly focuses on Pt based materials due to their noble character [6,7], or among others, Ir or Ir-based alloys [8,9]. Since Pt has a strong tendency to agglomerate, oxide dispersion hardening (ODS) was applied to stabilize the films [10]. Another alternative are ODS stabilized Mo films [11], because of the very high melting temperature of Mo, and therefore expected low creep at the operation temperature of the devices (600–800 °C). In our group, during the last years, many efforts were made to improve the high temperature stability of RuAl thin films. RuAl is a promising material due to its high melting temperature of 2050 °C [12] and its strong oxidation and corrosion resistance [13]. Recently, we realized RuAl thin films that were stable up to 800 °C in air and 900 °C in HV for at least 10 h [14].

The mass of the metal used for electrodes determines the velocity of the propagating SAW, and, thus, the coupling coefficient. Furthermore, heavy metals are especially used for reflective electrodes as, for instance, in resonator structures in the filter segment of SAW technology. In contrast, in other applications of SAW technology, e.g., in sensors, SAW-driven microfluidic actuators or SAW-based tags, where delay lines or reflective delay lines are often used, a more lightweight metal is often preferred to reduce the reflection of the acoustic energy at each of the finger electrodes.

Therefore, a wide range of mass loading provided by the metals used for the finger electrodes is essential to enhance the range of application in SAW technology. The above-listed materials, on which current research for high temperature metallizations focuses, all possess a high density (e.g., Pt: 21.45 g/cm${}^{3}$; Mo: 10.28 g/cm${}^{3}$; and RuAl: ≈8 g/cm${}^{3}$). In contrast to this, Ti-Al has a much lower density (3.9–4.3 g/cm${}^{3}$ [15]), which allows different design variations and with this additional applications of the devices.

For the very thin Ti-Al films (few 100 nm), the realization of the high temperature stability is more challenging than for the thick coatings or bulk materials. In the latter, during heat treatment at elevated temperatures, Al is oxidized and a several μm-thick protective oxide scale is formed on top of the sample. As a consequence, a thin Ti-Al film is destroyed completely under these conditions. Therefore, an additional barrier layer on top of the Ti-Al film is required to prevent oxidation. In this work, we applied SiO${}_{2}$ as a cover layer material, since it successfully protected RuAl thin films from oxidation up to 800 °C in air for at least 10 h [14,16]. A cover layer of Al${}_{2}$O${}_{3}$ was tested in the former work for RuAl thin films as well; however, it led to a stronger oxidation of the RuAl film as compared to SiO${}_{2}$ [16].

This paper presents a study on very thin (200 nm) Ti-Al films with focus on the high-temperature stability as well as on the γ-TiAl phase formation. Co-sputtered Ti-Al films with and without SiO${}_{2}$ protection layer were compared with multilayered Ti/Al films with SiO${}_{2}$ cover.

## 2. Materials and Methods

Ti-Al thin films with a thickness of 200 nm were prepared on Si substrates with 1 μm of thermally grown SiO${}_{2}$ on top by two different routines using DC sputtering. On the one hand, co-sputtering from elemental Ti and Al targets (100 mm target, $3.5\times 10^{-3}$ mbar, 500 W for Ti and 280 W for Al) was applied to deposit Ti-Al alloy films with a composition of 50:50. On the other hand, multilayer systems were prepared by subsequent sputtering of Ti and Al layers ($3.2\times 10^{-3}$ mbar, 500 W). The multilayer (ML) stacks were started with a Ti layer so that the uppermost layer consisted of Al. Two ML systems with a thickness of the individual layers of 10 or 20 nm were deposited. Since the total thickness of the film was kept constant at 200 nm, the Ti${}_{10\mathrm{nm}}$Al${}_{10\mathrm{nm}}$ bilayer was repeated 10 times and the Ti${}_{20\mathrm{nm}}$Al${}_{20\mathrm{nm}}$ 5 times. A 20-nm-thick SiO${}_{2}$ cover layer (RF sputtering, 200 mm target, $3.5\times 10^{-3}$ mbar, 2000 W) was added on top of some of the samples as an oxidation barrier. The SiO${}_{2}$ films were deposited by sputtering from a SiO${}_{2}$ target at a deposition temperature of 180 °C with a sputtering gas of Ar and O${}_{2}$ (ratio of 6:1). The depositions of the metallization and the cover layers were carried out in different chambers of the same cluster tool (CREAMET 350-CL 6, CREAVAC-Creative Vakuumbeschichtung GmbH, Dresden, Germany) without interruption of the high vacuum.

The films were annealed in high vacuum (HV, pressure lower than $10^{-5}$ mbar) for 10 h at 400, 600, and 800 °C. X-ray diffraction in Bragg Brentano geometry (XRD, Philips X’pert, Co Kα) was applied to analyze the phase formation. To reveal the full texture information and to confirm the formation of the γ-TiAl phase, pole figure measurements of the (101) and (110) lattice planes were performed (Philips X’Pert MRD, Cu Kα). For the γ-TiAl phase, the diffraction angle 2θ is 38.7° for the (101) and 45.3° for the (110) pole. To realize an optimum quality of these analyses, prior to each measurement, overview scans were performed with this theoretical value to identify the respective pole. Then, for each sample, the 2θ value was measured at this position, since it might slightly deviate from the theoretical value due to a small change of the composition or due to stresses in the thin film during growth and subsequent annealing. This optimized 2θ value was used for the texture measurement. The theoretical peak position was used if it was not possible to identify a pole with the overview scan.

Cross sections of the samples were prepared by the focussed ion beam technique (FIB) and imaged by scanning electron microscopy (SEM) in the same device (Zeiss 1540 XB Cross Beam, Carl Zeiss Microscopy GmbH, Oberkochen, Germany).

The distribution of the elements across the film thickness was analyzed by Auger electron spectroscopy (AES, JEOL JAMP-9500 Field Emission Auger Microprobe). Ar sputtering (energy of Ar ions: 1 keV, current of $0.7\times 10^{-6}$ A) was done for a time span between 30 and 120 s, and, subsequently, the AES spectra were recorded. These processes were repeated until the substrate was reached. The evaluation of the spectra was carried out using standard single element sensitivity factors of the PHI-Multipak software [17]. The analysis of the AES peak shape and position additionally allows deriving conclusions on the oxidation state of the metal elements.

For selected samples, X-ray photoelectron spectroscopy (XPS, PHI 5600 CI-System, Physical Electronics) was performed. Non-monochromatic MgKα X-rays (400 W) were used to excite the sample and a hemispherical electron analyzer working at a pass energy of 29 eV was applied to record the spectra. XPS allows a more detailed analysis of the oxidation state of the elements. In this case, depth profiles were also obtained by alternately sputtering with Ar ions (3.5 keV) and measuring the spectra.

Annular dark field scanning transmission electron microscopy (ADF-STEM, Technai F30, FEI company, Hillsboro, OR, USA) was used to image the local morphology. The local composition was determined by energy dispersive X-ray spectroscopy in the TEM (Octane T Optima, EDAX Company, Mahwah, NJ, USA). The TEM lamella were prepared using the FIB technique.

## 3. Results and Discussion

### 3.1. Phase Formation

**Figure 1:**
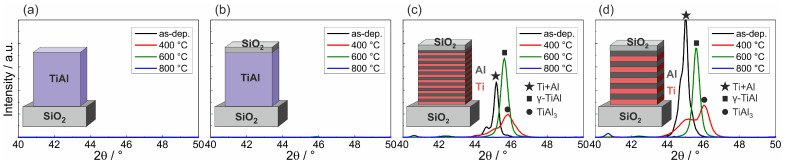
XRD measurements on: (**a**) a co-sputtered Ti-Al film; (**b**) a co-sputtered Ti-Al film with SiO${}_{2}$ cover layer; (**c**) a Ti/Al ML film with 10 nm individual layer thickness; and (**d**) a Ti/Al ML film with 20 nm individual layer thickness, both with SiO${}_{2}$ cover layer.

Figure 1 presents the results of the XRD measurements of the different samples in the as-prepared state and after annealing at 400, 600, and 800 °C in HV. It can be seen that for both co-sputtered films no peaks were visible for all sample states (Figure 1a,b). In contrast to this, clear XRD peaks were present for the ML films. In the as-prepared state, a superposition of the Ti (002, theoretical peak position: 2$\theta =44.96^{{}^{\circ}}$) and Al (111, theoretical peak position: 2$\theta =45^{{}^{\circ}}$) appeared (Figure 1c,d). The measured peaks were slightly shifted with respect to the theoretical position, which might be due to stresses in the thin films. Annealing at 400 °C led to a decrease of the peak intensities. However, there was still a superposition of two XRD peaks.

The phase formation in μm thick Ti/Al ML films was analyzed by various authors. For very thin individual layers (2 nm), a transition from (Ti) + (Al) → disordered TiAl + (Ti) →γ-TiAl + α2-Ti${}_{2}$Al was described by Ramos et al. [18]. In contrast to this, for thicker individual layers (100 nm), the authors described the transition from Al and Ti to γ-TiAl via a TiAl${}_{3}$ intermediate phase. The occurrence of TiAl${}_{3}$ in the phase sequence during annealing of Ti/Al ML samples was also described by Illekova et al. for an individual layer thickness of 20 nm or more [5]. For Al rich Ti-Al ML samples (substrate/40 nm Ti/180 nm Al/40 nm Ti/180 nm Al), the formation of TiAl${}_{3}$ was also observed after a heat treatment at 450 °C [19,20]. The two peaks measured in our work after annealing at 400 °C are therefore ascribed to Ti (002, now measured at a slightly higher 2θ of 45.1°) and TiAl${}_{3}$ (103 or 112, theoretical $2\theta =45.82^{{}^{\circ}}$, measured at 46.0°).

After annealing at 600 °C, one single peak was measured for both ML samples, which can be attributed to the γ-TiAl (101) peak (theoretical position: $2\theta =45.27^{{}^{\circ}}$, measured at 45.6°). After annealing at 800 °C, the TiAl peak disappeared.

**Figure 2:**
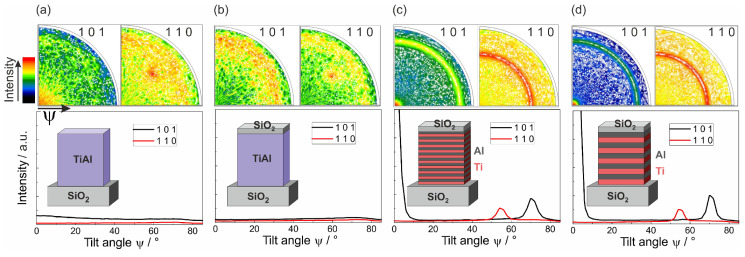
Results of the texture measurements of: (**a**) a co-sputtered Ti-Al film; (**b**) a co-sputtered Ti-Al film with SiO${}_{2}$ cover layer; (**c**) a ML Ti/Al film with 10 nm individual layer thickness; and (**d**) a ML Ti/Al film with 20 nm individual layer thickness, both with SiO${}_{2}$ cover layer, after annealing at 600 °C. The graphs represent the azimuthally averaged intensity versus the tilt angle Ψ.

Pole figure measurements were performed to confirm the formation of the γ-TiAl phase in the ML films. In addition, these measurements allowed to exclude that in the co-sputtered films the γ-TiAl phase has formed with a tilted texture, which cannot be detected by XRD in Bragg Brentano geometry. Figure 2 summarizes the pole figures of the (110) and (101) γ-TiAl poles of the four different samples annealed at 600 °C for 10 h. Since the pole figures are scaled to their respective maximum, in addition, the azimuthally averaged intensity versus the tilt angle Ψ is presented to allow a comparison between the different samples. It can be seen that both co-sputtered samples with or without SiO${}_{2}$ cover layer did not show any phase formation (see Figure 2a,b). The pole, which was visible in the TiAl (110) pole figure at $\Psi =54^{{}^{\circ}}$ was caused by the Si (220) lattice planes. Their 2θ value of 47.3° (Cu Kα) is close to that of the TiAl (110), so that it is also measured. Besides this, no significant intensity was detected.

In contrast to this, both ML samples showed a strong intensity in the center of the (101) pole figure and a clear ring at $\Psi =72^{{}^{\circ}}$ (see Figure 2c,d). Corresponding to this, the TiAl (110) pole figure contained a ring at $\Psi =54^{{}^{\circ}}$. These two measurements confirmed the formation of the γ-TiAl phase.

It is known from the literature that Ti-Al films co-sputtered at room temperature are in general an amorphous mixture of Ti and Al [4,21,22]. The formation of crystalline TiAl phases in amorphous Ti-Al or multilayer Ti/Al thick films in the range of 1.6 μm to 150 μm was investigated by several authors. Illekova et al. studied the activation energy of the phase formation in Ti/Al multilayers with various thicknesses of the individual layer (from 4 to 1000 nm). For a thickness of the individual layer of 20 nm, they derived a value of 169±3 kJ/mol [5]. They also described that for very thin individual layers (4 nm) at first the material becomes amorphous, so that the start of the crystallization is delayed. Senkov et al. analyzed amorphous Ti-Al films and determined an activation energy for the transition from the amorphous state to the first crystalline phase of 315±5 kJ/mol [23]. Although these values were extracted for films with a much larger thickness, the higher activation energy for the formation of the crystalline TiAl phase in amorphous films as compared to ML films can explain the observed difference in the behaviour of the thin films studied in this work.

### 3.2. Film Morphology

**Figure 3:**
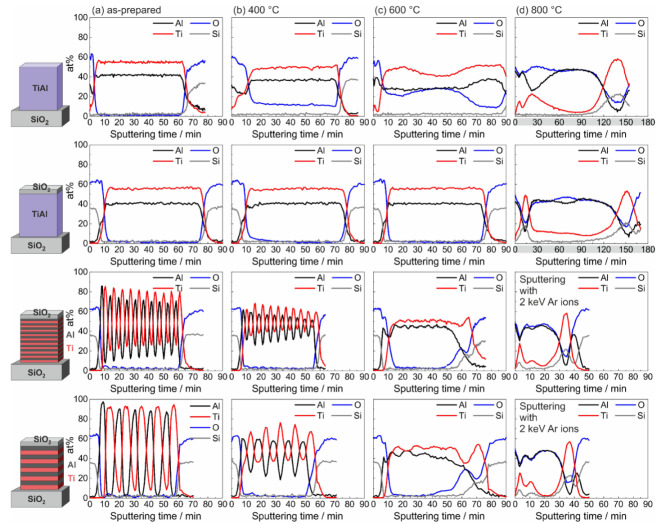
Results of the AES measurements of the co-sputtered Ti-Al film, co-sputtered Ti-Al film with SiO${}_{2}$ cover layer, ML Ti/Al film with 10 nm and 20 nm individual layer thickness, both with SiO${}_{2}$ cover layer in: (**a**) the as-prepared state; (**b**) after annealing at 400 ${}^{{}^{\circ}}$C; (**c**) after annealing at 600 °C; and (**d**) after annealing at 800 °C. In (**d**), the gray contrast of the abscissa in the figures of the alloy samples points out the longer sputtering time as compared to the other graphs. In contrast to this, the sputtering of the ML samples was performed with a higher sputtering energy (2 keV instead of 1 keV), which resulted in a shorter total sputtering time.

Figure 3 shows the results of the evaluation of the measured AES depth profiles for the amorphous and ML Ti-Al samples in the as-prepared state and after annealing at 400, 600, and 800 °C in HV. The composition was calculated from the measured AES spectra using the standard single element relative sensitivity factors (RSF). As can be seen for the co-sputtered films in the as-prepared state (Figure 3a,b), the calculation led to a higher atomic concentration of Ti. In contrast to this, an EDX analysis of a thin lamella of such a sample in the TEM resulted in an atomic composition with slightly more Al than Ti. This deviation of the results can be explained by different actual values of the RSF for the Ti-Al alloy and by a preferential sputtering. The AES results therefore mainly allow a relative comparison of the different samples and annealing states. Besides this, from the analysis of the peak shape and position, the oxidation state of Ti, Al, and Si was derived.

In Figure 3a, the results of the measurements of the samples in the as-prepared state are presented. In the uncovered co-sputtered film, a thin Al${}_{2}$O${}_{3}$ layer formed at the sample surface, which can be explained by the lower free energy of the formation of Al oxide as compared to that of TiO${}_{2}$ for pure elements [24]. (It is reported in the literature that in Ti-Al materials the oxide formation depends on the composition and the phases which are present in the material. Rahmel and Spencer determined that Al oxide is more stable than TiO${}_{2}$ in Al rich Ti-Al materials [25].) Across the whole sample thickness, the concentration of Al and Ti was constant.

In the SiO${}_{2}$ covered alloy film as well, a thin Al${}_{2}$O${}_{3}$ layer developed at the interface between the cover layer and the Ti-Al film. For both ML samples, the sequence of the Ti and Al layers was clearly visible. The concentration of both elements did not reach 100 at% in the separate layers and, especially for the film with 10 nm individual layer thickness, a decrease of the amplitudes of the element concentration with increasing sputtering time was seen. These findings can be explained by a partial intermixing of the elements at the interfaces during the sputtering process and by a roughening of the sample surface due to the Ar sputtering, which increases with increasing sputtering time. This roughening led to a simultaneous measurement of different depths, which resulted in an apparent mixture of Ti and Al. In addition, some intermixing can result from the high ion energy during the depositions of the layers. In these ML samples as well, Al${}_{2}$O${}_{3}$ was present below the SiO${}_{2}$ cover layer. The SiO${}_{2}$ layer was deposited at 180 °C. Obviously, this temperature is sufficient to initiate a reduction reaction of the SiO${}_{2}$ to Si by Al which forms Al${}_{2}$O${}_{3}$. This effect, which has been widely studied (see, e.g., [26]), was proven for these samples by XPS measurements.

The AES profiles of the samples after annealing at 400 °C in HV are shown in Figure 3b. For the uncovered co-sputtered sample, a strong oxide peak was visible at the sample surface, and a constant O signal was measured across the whole sample thickness. However, except for the sample surface, the AES showed Al and Ti only in the elemental and not in the oxidized state, which was derived from the AES peak position. This indicated that the O was solved within the amorphous Ti-Al layer. In contrast to this, in the co-sputtered film with SiO${}_{2}$ cover layer as well as in the ML films no O was measured within the sample. In the ML films, a partly interdiffusion of Ti and Al occurred. In addition, at the Ti-SiO${}_{2}$ interface at the bottom of the film some O was detected in the Ti layer.

After annealing at 600 °C, in the co-sputtered film without cover layer the O signal strongly increased. Within the first 5 min of sputtering, Ti was present in the oxidized and for the rest of the measurement in the elemental state. Oxidized Al was found at the sample surface. In the regime between 10 and 20 min of sputtering, Al was present in the elemental state, and the O which was detected there was again solved in the TiAl without forming an oxide. With increasing sputtering time, the amount of oxidized Al increased again, and all Al was present in the oxidized state for the sputtering time between about 35 and 50 min. Afterwards, the part of elemental Al increased again, and in the time regime between about 70 and 85 min only elemental Al was found. Again, O was just solved without forming an oxide. At this temperature, the SiO${}_{2}$ cover layer still successfully acted as a diffusion barrier in the case of the co-sputtered Ti-Al film, which was seen from the constant thickness of the Al${}_{2}$O${}_{3}$ layer below it. However, in the case of the ML samples, the thickness of the Al${}_{2}$O${}_{3}$ interlayer between the SiO${}_{2}$ cover and the TiAl layer increased. At this interfacial region as well, XPS proved the presence of elemental Si. The interdiffusion of the Al and Ti layers in the ML samples was almost completed and only a small variation of the composition remained visible. The annealing at 600 °C, however, led to a strong reaction in the Ti/Al ML samples with the SiO${}_{2}$ at the substrate surface. The measurements revealed a layer consisting of elemental Ti and Si on top of the substrate, followed by an Al${}_{2}$O${}_{3}$ layer.

The annealing at 800 °C (Figure 3d) led to a degradation of all films and all Al was oxidized. The formed Al${}_{2}$O${}_{3}$ was mainly present in the center of the film. For all samples, at the interface to the substrate a strong Ti and Si signal was measured. In the samples with SiO${}_{2}$ cover layer, Al${}_{2}$O${}_{3}$ was found at the surface of the film. In the case of the co-sputtered film, below this Al${}_{2}$O${}_{3}$, there was a defined layer consisting of Ti and Si. In the ML samples, Ti and Si were distributed across a broader thickness in the upper region of the film, and no oxidized Ti was found at all.

The time necessary to sputter the film increased significantly as compared to the previous measurements. One reason was the low sputter rate of Al${}_{2}$O${}_{3}$. In addition, due to the insulating material, electric charges were accumulated, leading to electric fields which further decreased the sputter rate. Therefore, for the ML samples an increased energy of the Ar ions (2 keV instead of 1 keV) was applied to achieve higher sputter rates.

The AES results showed that the chemical reaction between the Ti-Al film and the SiO${}_{2}$ cover layer and substrate strongly depended on the initial distribution of the elements Ti and Al in the film. In the ML samples, stronger reactions took place at the respective interfaces between the pure Al layer on top and the pure Ti layer at the bottom and the SiO${}_{2}$: a pure Ti layer at the bottom of the sample resulted in a marginal reaction between Ti and Si already at 400 °C (see the O signal in the bottom Ti layer) and a strong reaction at 600 °C. In contrast to this, in the case of the Ti-Al alloy, such a reaction did not take place at these temperatures. There was also a stronger oxidation of the Al at the interface with the SiO${}_{2}$ cover layer in case of the pure Al layer, as can be seen from the thicker Al${}_{2}$O${}_{3}$ interlayer.

**Figure 4:**
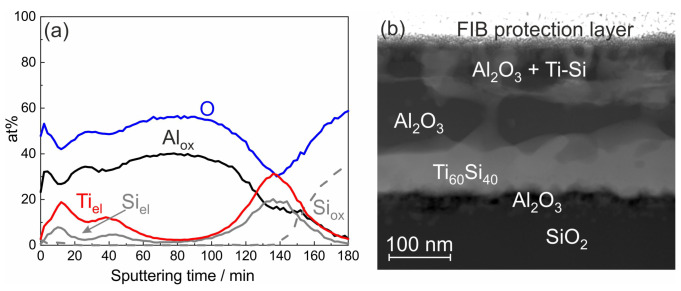
(**a**) Results of the XPS measurement of the ML sample with 20 nm individual layer thickness; and (**b**) STEM image with dominant chemical contrast, both after annealing at 800 °C in HV. In (**a**), the subscript “el” refers to the elemental and “ox” to the oxidized state of the respective element.

XPS was used to identify the chemical state of the elements in the degraded films. The result of an XPS depth profile analysis of the ML Ti/Al sample with 20 nm individual layer thickness and SiO${}_{2}$ cover layer after annealing at 800 °C is shown in Figure 4a. The measurements revealed that Al was only present in the oxidized state (marked by Al${}_{\mathrm{ox}}$), and the intensity curve follows that of O. Ti was only present in the elemental state (marked by Ti${}_{\mathrm{el}}$) and was not oxidized at all. Unoxidized Si was found in the upper and lower region of the film, where its intensity curve was parallel to that of Ti. This behavior indicated the formation of a Ti-Si phase.

In contrast to the ML samples, in the co-sputtered films, the O signal was not parallel to that of Al (see Figure 3d). Around the sputtering time of 30 min, the film without the cover layer showed a strong decrease in the Al, a slightly increasing O, and a maximum in the Ti signal. An analysis of the AES spectral shape revealed that in this region of the film a part of the Ti was oxidized. In the co-sputtered film with SiO${}_{2}$ cover as well, at the sputtering time around 70 min, a stronger O signal as compared to Al was visible. Again, a part of the Ti was oxidized, however, to a much reduced amount as compared to the sample without cover layer.

The reaction between Ti and SiO${}_{2}$ is widely described in the literature (see, e.g., [27,28]). There is a chemical reaction between Ti and SiO${}_{2}$ at their interface, leading to the formation of a Ti silicide. The free O diffuses through the Ti layer, which is possible because of the high O solubility of 34 at% in this material [29]. This solution of O in the bottom Ti layer was already visible for the ML systems after annealing at 400 °C (see Figure 3b). During annealing at 600 °C, the interface reaction became stronger, and the O which diffused through the Ti layer then reacted with the Al on top of it to Al${}_{2}$O${}_{3}$. Ti not only reacted with the SiO${}_{2}$ of the substrate, but also with the covering SiO${}_{2}$ layer, so that at the film surface Ti-Si was also formed.

In the upper region of the film, a small carbon signal of less than 5 at% was measured by XPS, indicating C which was present in a carbide state. However, most likely the carbide was formed by the Ar sputtering and did not originate from the sample. (More details on the XPS measurement of the Ti-Al samples were published by Oswald et al. [30].)

To get more information on the morphology of this sample, a STEM analysis was performed. A STEM image predominantly showing the chemical contrast is presented in Figure 4b. The EDX analysis confirmed the formation of Al${}_{2}$O${}_{3}$ and the presence of Ti-Si in the upper and lower region of the film. The chemical composition of the Ti-Si grains in the lower region of the film was about Ti${}_{60}$Al${}_{40}$. Close to this composition, there are the two Ti-Si phases: Ti${}_{5}$Si${}_{4}$ and Ti${}_{5}$Si${}_{3}$ [31]. There are reports describing the formation of the Ti${}_{5}$Si${}_{3}$ phase [27,28], and for our samples this phase was confirmed by the small Ti${}_{5}$Si${}_{3}$ (002) peak in the XRD data at $2\theta =40.6^{{}^{\circ}}$ in Figure 1c,d.

**Figure 5:**
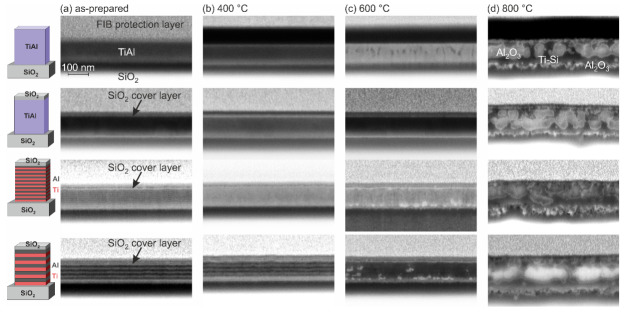
SEM images (inLens, 3 kV) of the FIB cross sections of the co-sputtered Ti-Al film, co-sputtered Ti-Al film with SiO${}_{2}$ cover layer, and Ti/Al ML film with 10- and 20-nm individual layer thickness, both with SiO${}_{2}$ cover layer in: (**a**) the as-prepared state; (**b**) after annealing at 400 °C; (**c**) after annealing at 600 °C; and (**d**) after annealing at 800 °C.

Figure 5 summarizes SEM images of the FIB cross sections of the different samples in the as-prepared state and after the annealing procedures. In the as-prepared state (Figure 5a), the co-sputtered films showed a homogeneous structure. It was challenging to resolve the 10 nm multilayers; however, the 20-nm Ti and Al layers were clearly visible (dark layers: Ti; brighter layers: Al). After annealing at 400 °C, the co-sputtered samples as well as the 10-nm ML film appeared homogeneous. In the 20-nm ML sample, it was still possible to identify the layer structure (Figure 5b).

Changes in the microstructure became visible in the co-sputtered film without cover layer after annealing at 600 °C. The SEM image showed that darker grains were present in the upper region of the film, and that there are no visible inhomogeneities in the lower region. The dark structures were most likely Ti rich grains. This is in agreement with the AES measurements (Figure 3c), which showed besides Al and O a strong Ti signal in the upper region of the film. In contrast to the uncovered film, the co-sputtered film with SiO${}_{2}$ layer did not possess such inhomogeneities as was expected from the AES measurements, which revealed a homogeneous composition. After annealing at 600 °C, in the images of both ML samples, small bright structures were present—in the case of the 10-nm ML in the lower region of the film and in the case of the 20-nm ML additionally in the center region of the TiAl layer. These bright structures were Al${}_{2}$O${}_{3}$ grains on top of the Ti${}_{5}$Si${}_{3}$ layer, which is in agreement with the AES results. In the 20-nm ML films, the AES showed a small O signal during the sputtering time between 40 and 55 min, which was not present in the 10-nm ML sample, caused by the Al${}_{2}$O${}_{3}$ grains that were distributed in the center region of the TiAl layer.

For all samples, strong changes in the morphology appeared after annealing at 800 °C (Figure 5d). Irregular formed structures were visible and the film thickness increased significantly. From the evaluation of the EDX data shown in Figure 4 in combination with the AES measurements, it can be derived that the large bright structures were Al${}_{2}$O${}_{3}$ grains. In the case of the uncovered film, these grains were embedded in a Ti and Ti-O matrix, and, in the case of the films with the SiO${}_{2}$ cover layer, the matrix consisted of Ti-Si. In all samples, the interface between the film and substrate was no longer clearly defined and showed a large roughness due to the reaction between Ti and SiO${}_{2}$.

## 4. Conclusions

This paper reports on the high temperature stability of very thin Ti-Al films. Co-sputtered or multilayered Ti-Al films with a thickness of 200 nm were deposited on thermally oxidized Si substrates, and annealed up to 800 °C in HV. Subsequently, they were analyzed regarding their phase formation and degradation processes up to 800 °C. In addition, the influence of a SiO${}_{2}$ cover layer was evaluated.

The results show that already after annealing at 400 °C co-sputtered Ti-Al films without cover layer started to oxidize. In contrast to this, films with a 20-nm-thick SiO${}_{2}$ cover layer were stable up to 600 °C. XRD measurements revealed that in the ML films the γ-TiAl phase formed during annealing at 600 °C. For the co-sputtered films, no phase formation was observed at all.

During annealing at 600 °C, in the ML samples, a chemical reaction between the Ti and SiO${}_{2}$ of the substrate, but also with the SiO${}_{2}$ cover layer, took place. This reaction led to the formation of Ti${}_{5}$Si${}_{3}$. The free O was solved in the TiAl layer and locally led to the formation of Al${}_{2}$O${}_{3}$. All films were destroyed during annealing at 800 °C. Ti silicide was formed to a large extent, and all Al, as well as in the film without cover layer some Ti, was oxidized.

In summary, the results reveal that the deposition of Ti/Al MLs is a prerequisite to achieve the γ-TiAl phase in the very thin films. In contrast to the μm thick TiAl films, which are described in literature, for the thin films, the chemical reaction between the Ti and SiO${}_{2}$ plays a crucial role. On the one hand, the part of the Ti that is chemically bonded to the Si is not available for the TiAl phase formation. On the other hand, the O that is emitted by this reaction leads to the formation of Al${}_{2}$O${}_{3}$ at 600 °C within the 10 h of annealing. This effect will be more pronounced for longer annealing times and higher temperatures, and with this contributes to the degradation of the film. For thick films, these processes are negligible, since they only affect a very small part of the total layer. The results also show that a SiO${}_{2}$ cover layer improves the high temperature stability of the TiAl films as compared to samples without cover layer despite the chemical reaction between Ti and SiO${}_{2}$.

To realize a high temperature stability of γ-TiAl thin films, a contact to SiO${}_{2}$ needs to be avoided. This could be achieved by substituting the barrier with another material, e.g., AlN, or by using an additional protection layer. For the application in SAW devices, a high temperature stability of TiAl thin films on other piezoelectric oxide materials such as CTGS substrates also has to be realized.

## Abbreviations

The following abbreviations are used in this manuscript:

AES	Auger electron spectroscopy
EDX	energy dispersive X-ray spectroscopy
FIB	focussed ion beam
HV	high vacuum
ML	multilayer
SAW	surface acoustic wave
SEM	scanning electron microscopy
STEM	scanning electron transmission microscopy
XPS	X-ray photoelectron spectroscopy
XRD	X-ray diffraction
